# Sensitivity Enhancement of Tube-Integrated MEMS Flow Sensor Using Flexible Copper on Polyimide Substrate

**DOI:** 10.3390/mi14010042

**Published:** 2022-12-24

**Authors:** Tsuyoshi Tsukada, Ryusei Takigawa, Yoshihiro Hasegawa, Muhammad Salman Al Farisi, Mitsuhiro Shikida

**Affiliations:** Department of Biomedical Information Sciences, Hiroshima City University, Hiroshima 731-3194, Japan

**Keywords:** MEMS, flow sensor, Cu on polyimide substrate, sensor integration

## Abstract

A tube-integrated flow sensor is proposed in this study by integrating a micro-electro mechanical systems (MEMS) flow-sensing element and electrical wiring structure on the same copper on polyimide (COP) substrate. The substrate was rolled into a circular tube with the flow-sensing element installed at the center of the tube. The signal lines were simultaneously formed and connected to the Cu layer of the substrate during the fabrication of the sensing structure, thus simplifying the electrical connection process. Finally, by rolling the fabricated sensor substrate, the flow sensor device itself was transformed into a circular tube structure, which defined the airflow region. By implementing several slits on the substrate, the sensing element was successfully placed at the center of the tube where the flow velocity is maximum. Compared to the conventional sensor structure in which the sensor was placed on the inner wall surface of the tube, the sensitivity of the sensor was doubled.

## 1. Introduction

Micro-electro mechanical systems (MEMS), which are realized by the semiconductor micro- and nano-fabrication technology that enables nano-level processing of minute mechanical structures, are able to provide ultra-compact and multifunctional devices. Today, many MEMS sensors, such as pressure sensors, accelerometers, and gyro-sensors, are indispensable in our daily lives and are used in various fields, such as automobiles, smartphones, and wearable devices. Traditionally, MEMS sensors have been fabricated based on Si materials [1]. This can be attributed to the excellent mechanical properties of Si and its processability by the semiconductor microfabrication technology [2].

How widespread MEMS is can also be attributed to mature back-end processing technologies, such as wire bonding and solder reflow. Such technologies have been readily applicable to Si-based MEMS sensors. In addition to those traditional techniques, some techniques aiming for integration of MEMS devices and other semiconductor devices have been developed. For instance, the development of a through silicon via which allows vertical electrical interconnection has led to the manufacturing of multi-layered devices [3,4]. Wafer bonding technology is another key technology that has allowed the three-dimensional stacking of heterogeneous devices at the wafer-level and device encapsulation simultaneously [5,6]. A customizable wide choice of materials for wafer bonding, from metallic materials [7], glasses [8] to polymers [9], has led to the practical application of wafer-level packaging technology being widespread [10,11,12].

With the development of materials sciences and mechanical engineering, a wide variety of materials have been implemented in MEMS. In particular, MEMS devices using polymer and composite materials have a potential to rival the traditional MEMS devices made of Si materials due to their excellent properties, such as low cost, flexibility, and biocompatibility [13,14]. For instance, intraocular pressure sensors for glaucoma monitoring [15], intravascular shear stress sensors to investigate the interplay between hemodynamics and arterial plaque formation [16], and flexible tactile sensors [17], have been developed using polymer materials. Innovative fabrication methods taking the advantage of the flexible materials have also been proposed. For instance, a sensor structure was fabricated by folding the substrate like origami [18], and a 3D structure using a substrate consisting of a metal and resin substrate bonded together have also been proposed [19].

MEMS thermal flow sensors have been traditionally fabricated using Si [20,21]. Due to the high demand of flexible sensors, thin polymer materials have recently emerged as substrate materials to form thermal flow sensors. Two types of MEMS thermal flow sensors have been widely utilized, namely hot-wire anemometers and calorimeters. Hot-wire anemometers utilize the dependence of the heat transferred from a heater working as a flow-sensing element on the substrate to the surrounding fluid on the airflow velocity. Meanwhile, calorimeters measure the flow velocity from the change in the heat distribution around the heater due to the airflow. Free-standing structures are often implemented in the Si-based flow sensors to limit the thermal capacity and maximize the sensor sensitivity and response characteristic. To achieve the same feature in flexible sensors, the sensing structure has been fabricated on a thin polymer substrate [22,23,24]. The flexible sensor structure made of polymer material can be easily mounted in a circular tube or on the wing surface. A series of flexible sensors using hydrogel as a conductive and stretchable material has also been proposed [25,26,27]. These can be fabricated by printing with a fine nozzle or through optical fabrication, where the hydrogel can be molded into arbitrary shapes. However, challenges still remain to implement this technology for MEMS flow sensor, where the conductive and insulating portions must be formed precisely.

Our research group has fabricated a micromachined tube-type flow sensor by forming a gold-sensing element on a thin polyimide substrate and mounting it inside a small tube [28,29]. However, some challenges remained in the mounting process since there is no general packaging technique applicable for flexible sensors [30]. On the other hand, it is practically challenging to implement the packaging technologies developed for Si sensors, which have been developed based on the semiconductor microfabrication technology, to the flexible sensors fabricated using polymer substrates. Manual insertion of the substrate into the tube package and thermo-compression bonding for electrical wiring connection was required for each sensor, which was labor-intensive in terms of the time, effort and complexity.

**Figure 1:**
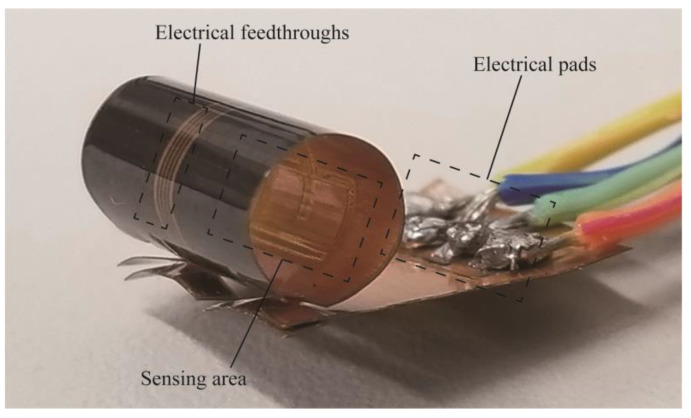
Photograph of a tube-type flow sensor previously fabricated using COP substrate. The flow measurement element was installed on the inner wall of the tube.

A MEMS fabrication method using a copper on polyimide (COP) substrate has been proposed to simplify the sensor mounting process [31]. The COP substrate consists of a thin copper layer laminated on a polyimide film. The thin copper layer can be used as embedded wiring for the sensing element by forming the sensor structure on the COP substrate. Then, the plastic deformation of the copper layer at the COP substrate can be utilized to maintain the deformed shape. By forming the sensor device and electrical wiring structure simultaneously on the same COP substrate, the process of connecting the fine wiring on the sensor substrate to external wiring substrates can be eliminated. In the previous study, a COP flow sensor has been proposed, in which the sensor device, a tube, and electrical connection substrate were integrated through the plastic deformation of a part of the sensor substrate to form a tube structure, as shown in Figure 1. However, the sensor had a limited sensitivity, because the sensing element was installed on the inner wall of the tube [32]. In this study, the position of the sensor is optimized by computational fluid dynamics (CFD) simulation. Based on the simulation results, a sensor structure and fabrication method are proposed to place the sensing element at the optimized position.

## 2. Fluid Analysis of Conventional and Proposed Flow Sensor

In our previous work, we fabricated a MEMS flow sensor based on COP substrate with an integrated tube structure as shown in Figure 1. A flexible COP substrate was utilized to fabricate the sensing structure and electrical wiring simultaneously on the same substrate. The substrate was then transformed into a tube structure by rolling it into a cylindrical shape. The flow-sensing element was placed on the tube inner wall and the airflow measurement using the tube-shaped sensor was demonstrated. The fine wirings of the sensing element were directly connected to larger electrical pads on the substrate, from which the electrical wiring can be further extended using conventional solder. In the photograph in Figure 1, the electrical pads are very large in order to facilitate the evaluation by manual wiring connection using solder. The size of the electrical pads can be adjusted in accordance to the purpose by changing the mask pattern in the fabrication process. It is also possible to make the electrode pads smaller and connect them to another flexible printed circuit with anisotropic conductive paste. The integration of the driving circuit was also demonstrated on the COP substrate [33]. In this study, a CFD simulation was performed to analyze the optimum place for the sensing element.

**Figure 2:**
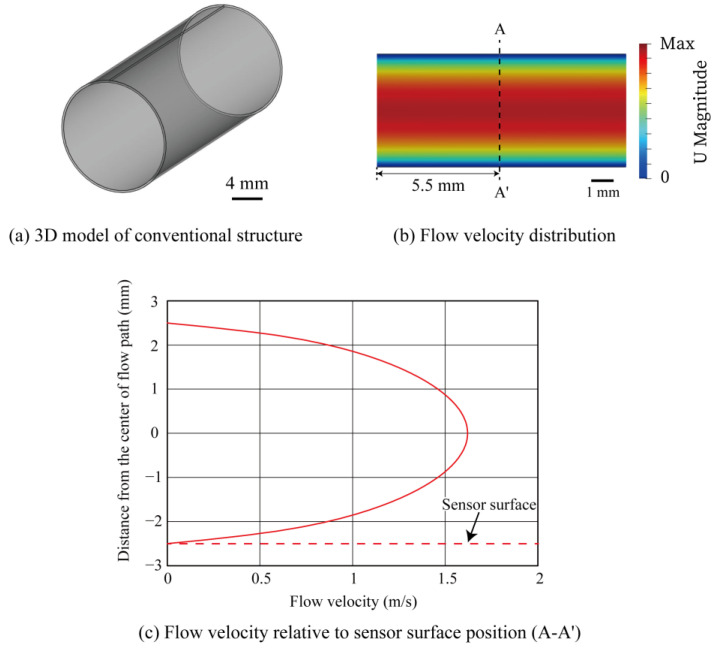
Fluid analysis in the conventional structure. The sensing structure was installed on the wall of the circular tube (at −2.5 mm).

OpenFOAM, an open-source fluid analysis software, was utilized to simulate the fluid flow in a circular tube. The analysis conditions are shown below and in Table 1. The channel length and inner diameter of the tube were 11 mm and 5.0 mm, respectively. The flow analysis procedure is described as follows. First, a 3D tube model was created using FreeCAD, an open-source 3D modeling software. The circular tube model of conventional sensor was designed as depicted in Figure 2a.

Next, the 3D model was represented by grid meshes using OpenFOAM’s standard utilities, blockMesh and SnappyHexMesh. The total number of meshes was set to 150,000. The CFD analysis was performed with the boundary conditions of 1.0 m/s inlet flow velocity into the tube and static pressure at the tube outlet. The analysis was performed using OpenFOAM’s simpleFOAM solver. The analysis results were visualized using paraView. The simulation result of the flow inside the circular tube is visualized as shown in Figure 2b. Figure 2c shows the simulated flow velocity as a function of the radial axis across the tube. The sensor surface indicates the mounting location of the flow-sensing element. According to the simulation result, the flow velocity was the lowest near the inner wall, which was the installation place of the sensing element in the conventional design (red dotted line). The flow velocity increased as it moved toward the center of the tube. In agreement with the Hagen–Poiseuille law, when the flow inside a circular tube is laminar, the flow velocity distribution inside the tube generally follows a rotating parabolic plane, with zero flow velocity at the tube wall and maximum velocity at the center of the tube.

**Figure 3:**
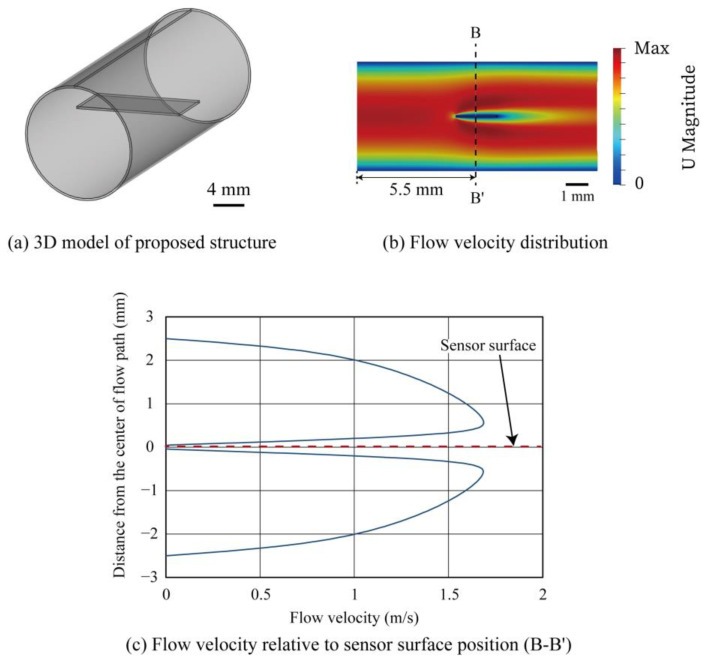
Fluid analysis with the proposed structure. The sensing structure was installed on a plate in the center of the circular tube (at 0 mm position).

**Figure 4:**
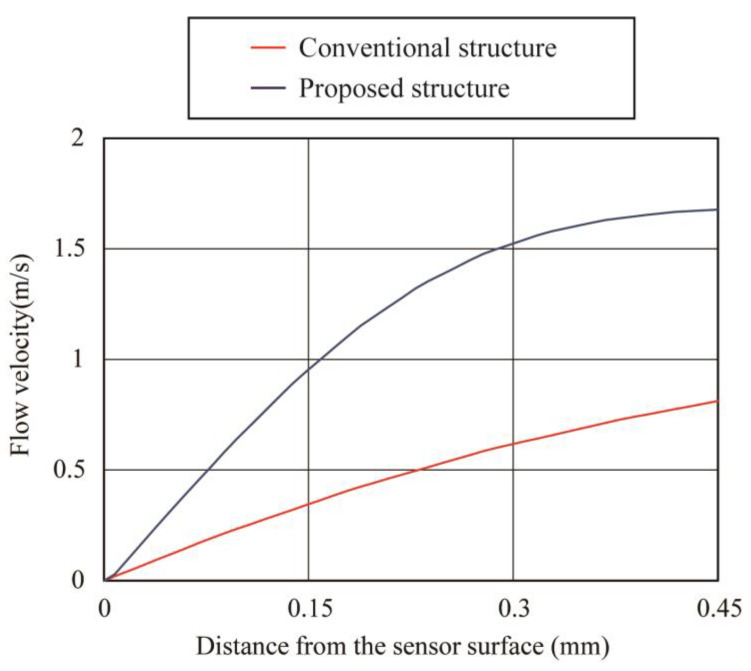
Flow velocity versus distance from sensor surface for conventional and proposed structures. The proposed structure with the sensor placed in the center of the tube has a large slope of flow velocity change.

In the conventional MEMS flow sensor structure based on COP, the sensing element was fabricated on the COP substrate, which was then rolled into a cylindrical shape. Through such a process, the flow-sensing element was installed on the tube wall surface where the flow velocity was the slowest in the tube. Therefore, the sensor sensitivity was limited. According to the above simulation results, a new structure with the flow-sensing element placed at the center of the tube is proposed as depicted in Figure 3a. The result of the CFD analysis of the proposed structure is shown in Figure 3b. The boundary conditions were maintained the same as the previous simulation. Figure 3c shows the relation of the flow velocity and the radial position near the sensing element. The sensor was placed at 0 mm on the vertical axis. The flow velocity is zero on the plate where the sensing element is installed because the plate containing the sensing element disturbs the flow path. The flow velocity on the plate where the sensing element is placed is 0 m/s; however, the flow velocity increased significantly as it moves away from the plate to the maximum value at the distance of around 0.55 mm above the surface of the sensing element. Since the flow sensor works as a hot-wire anemometer that measures flow based on the thermal principle (see below for the measurement principle), the flow velocity is measured using the heat convention at the vicinity of the surface of the sensing element. As a consequence, a higher sensitivity can be expected due to the greater slope of the flow velocity relative to the distance from the surface of the sensing element. Figure 4 shows the comparison of the relationship between the distance from the surface of the sensing elements and the flow velocity extracted from the graphs in Figure 2c and Figure 3c. According to the simulation results, the slope of the flow velocity was larger for the structure with the flow-sensing element installed in the center of the tube; therefore, a higher sensitivity can be expected.

## 3. Sensor Structure and Detection Principle

**Figure 5:**
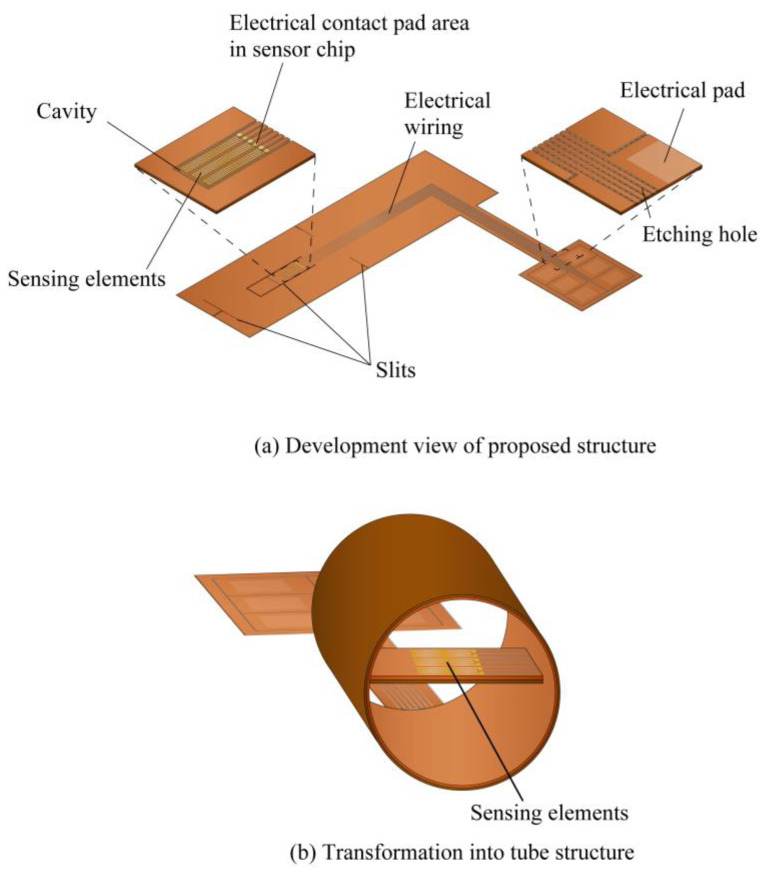
Schematic of the proposed flow sensor. Development of the sensing element fabricated on a COP substrate (**a**). The substrate deformed into a tube structure (**b**).

**Figure 6:**
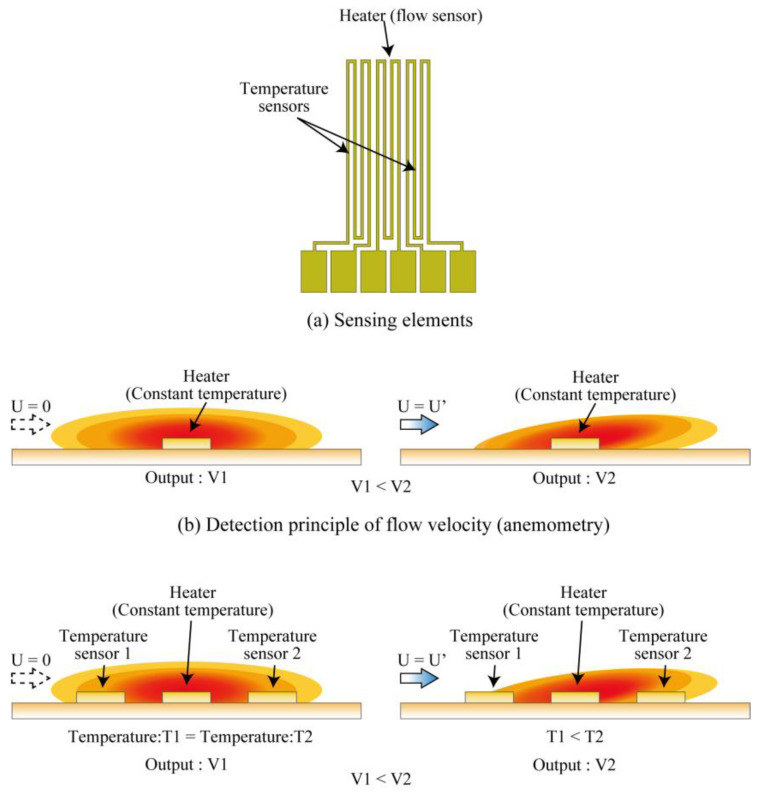
Sensing element patterns and principles of flow velocity (anemometry) and flow direction measurement (calorimetry).

Based on the results of CFD simulation, a new COP flow sensor is proposed with the sensing element placed at the center of the tube. Figure 5 illustrates the development view and the assembled view of the sensor. The flow-sensing element, embedded electrical wiring, and connection pads are formed on the COP substrate. A cavity is provided for thermal insulation underneath the sensing element. The sensor substrate is slit, rolled and assembled to form the flow sensor device with the sensing element placed at the center of the tube as illustrated in Figure 5b. The flow-sensing element consists of a heater working as the flow sensor and 2 temperature sensors each on the upstream and the downstream directions of the heater to identify the flow direction as shown in Figure 6a. The flow-sensing element was fabricated using a Au thin film.

The proposed flow sensor measures the airflow through the tube by using the metal heater (resistive element) as a hot wire anemometer as illustrated in Figure 6b. The heater was externally incorporated in a Wheatstone bridge and driven by a constant temperature driving feedback circuit combined with an integrating circuit. When the circuit is driven, a driving voltage is applied to the heater and the heater generates heat. The heating temperature is controlled by adjusting the voltage applied to the heater using a variable resistor in the Wheatstone bridge. When an airflow passes through the heater that is operating at a certain temperature, the heat from the heater is lost to the airflow and its temperature drops. Since the resistance of a metal heater changes with the temperature, the equilibrium of the Wheatstone bridge is disrupted by the drop in temperature of the heater. In such a case, the integrating circuit operates to maintain the equilibrium in the Wheatstone bridge by supplying extra voltage. As a result, the heater heats up and returns to its original set temperature (resistance), thus maintaining the equilibrium of the bridge. Taking advantage of such a correlation between the amount of the electrical energy consumed by the heater and the airflow velocity, the sensor can measure the flow rate through the tube [34,35].

The proposed sensor is also equipped with a flow direction detection function by using temperature sensors installed on both the upstream and downstream sides of the heater. The airflow detection direction mechanism is illustrated in Figure 6c. In the absence of the airflow, the two temperature sensors are evenly heated by the heater. When the airflow passes through the heater, the heat from the heater is carried downstream by the airflow. As a result, the heat distribution of the heater is disrupted and a difference of readings between the temperature sensors is generated. The flow direction can be detected by measuring the difference in resistance between the temperature sensors caused by the disrupted heat distribution using a voltage divider circuit and a differential amplifier circuit.

## 4. Sensor Fabrication and Basic Characteristic Evaluation

**Figure 7:**
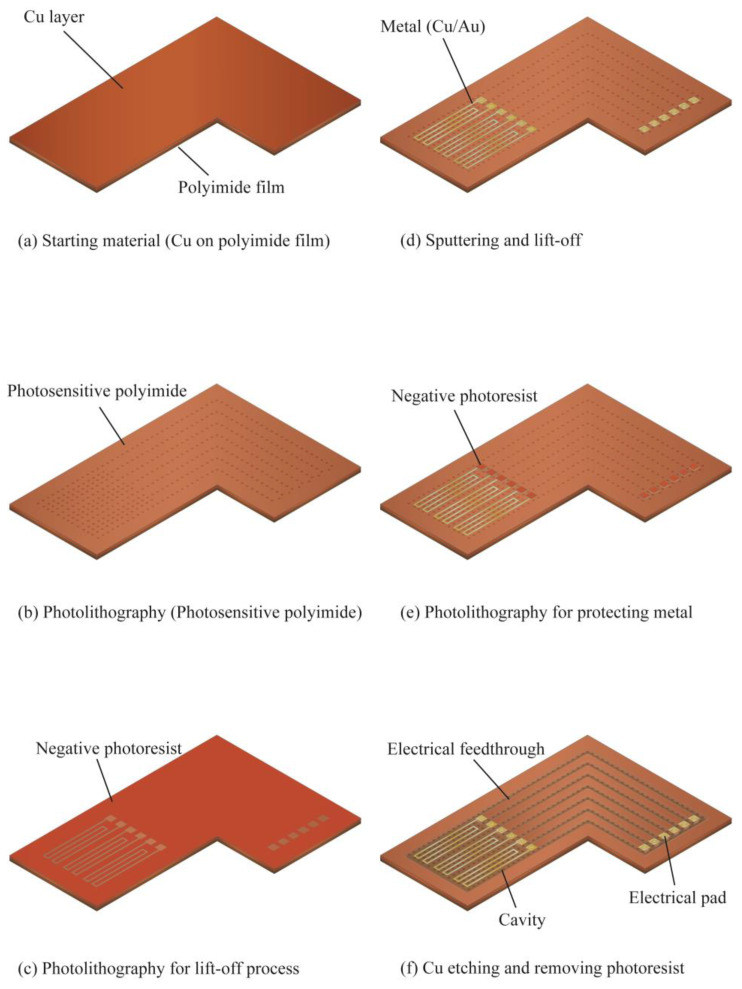
Fabrication process for sensing element on a COP substrate.

The fabrication process of the sensor is illustrated in Figure 7. A COP substrate (SR-1220, UBE EXSYMO CO., LTD., Tokyo, Japan) was used as the substrate material. The substrate consists of a 38 μm-thick Cu layer laminated on a 50 μm-thick polyimide film as illustrated in Figure 7a. A photosensitive polyimide (PW-1500, TORAY DUPON CO., LTD., Tokyo, Japan) was spin-coated on the Cu layer of the COP substrate. The thickness of the spin-coated photosensitive polyimide was 3.8 μm. Next, hole patterns were formed using the photosensitive polyimide layer by photolithography for selective sacrificial etching of the Cu layer in the final step, as shown in Figure 7b. Then, the spin-coated polyimide layer was cured in a thermostatic oven at 140 °C for 30 min and 260 °C for 60 min, subsequently. A negative photoresist (ZPN-1150, ZEON corporation) was further spin-coated and patterned through photolithography on top of the photosensitive polyimide layer. The 5 μm-thick pattern was used to form the pattern for the metal heater, temperature sensors, and electrode pads, as illustrated in Figure 7c.

Au and Cr thin films were then deposited on the patterned photoresist by sputtering, as shown in Figure 7d. The Cr was used as the adhesion promotion layer and the Au layer plays a role as the sensing element. The thickness of Au and Cr layers were 250 nm and 10 nm, respectively. Then, the metal patterns of the sensing element and electrical pads were formed by removing the photoresist using acetone solution (lift-off process). The electrical wiring from the sensing element was connected to the Cu layer via the holes pattern formed previously in the step indicated in Figure 7b. Then, another layer of negative photoresist (ZPN-1150) was spin-coated and patterned to selectively protect the electrical pads exposed in the previous step. In the following step, the copper layer was selectively etched from the holes formed indicated in Figure 7b. When the photoresist was spin-coated in this process, the resist also penetrated into those holes. To prevent etching defects, the photoresist in the holes must be completely removed. To prevent the photoresist in the holes from not being removed due to insufficient exposure, a negative type photoresist was selected for this process. Finally, a cavity for thermal insulation of the heater and electrical wiring were formed by selectively etching the Cu layer using ferric chloride solution at 30 °C through the holes formed in the photosensitive polyimide layer in step shown in Figure 7b. After the underlayer etching was completed, the protective photoresist in patterned in step shown in Figure 7e was removed with acetone solution. Finally, the sensor was cleaned with a de-ionized water, and dried after immersion in isopropyl alcohol.

**Figure 8:**
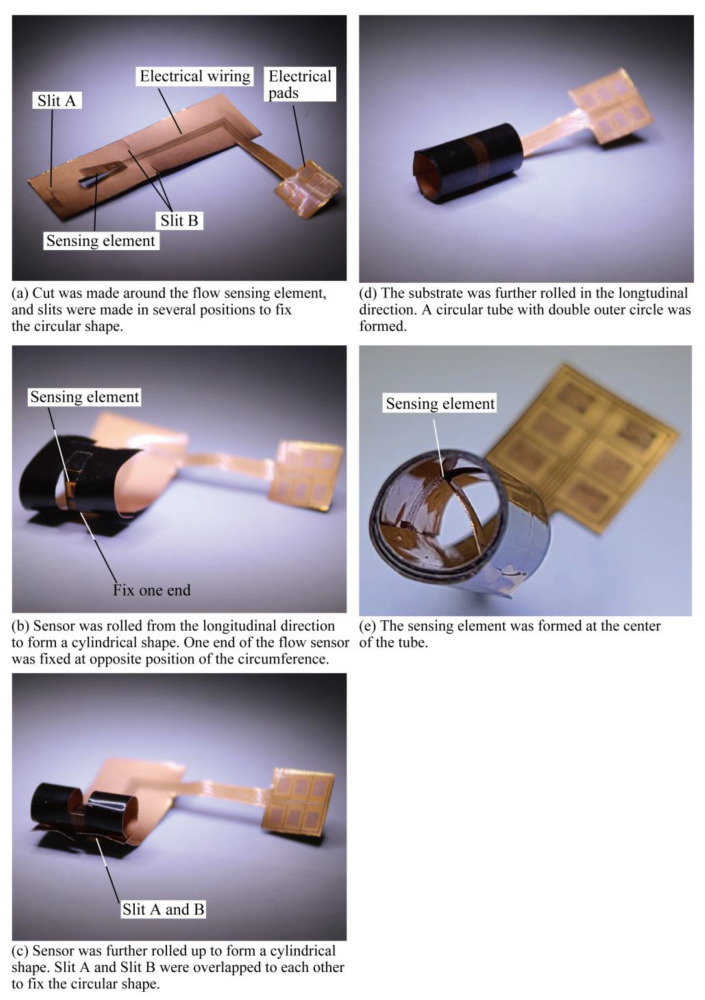
Procedure for deforming the sensor fabricated on the COP substrate into a circular tube shape.

The assembly process of the fabricated COP MEMS flow sensor is described as shown in Figure 8. First, the unit sensor was cut out from the substrate using scissors. Then, using a design knife, a cut was made around the flow-sensing element. To assist the creation of the tube structure, slits were also made in several positions, as shown in Figure 8a. Then, the sensor was rolled from the longitudinal direction to form a cylindrical shape as illustrated in Figure 8b. When a semicircular shape was formed, one end of the flow sensor part was fixed at the opposite position of the circumference using a polyimide tape. This step defined the position of the sensing element at the center of the tube which will be formed. Next, the sensor was further rolled up to form a cylindrical shape as shown in Figure 8c. After a full circle was formed, the slits formed in step indicated in Figure 8a were overlapped with each other to fix the circular shape. A hole remained at the cylindrical shape due to the cut around the sensing element produced in first step. To prevent airflow leakage due to the hole existing in the previous step, the substrate was further rolled in the longitudinal direction as illustrated in Figure 8d. After a circular tube with double outer circle layers was formed, the tube was fixed in the circular shape using polyimide tape. The circular shape was fixed and defined by the plastic deformation of the thin Cu layer and the polyimide tape. A photograph of the fabricated sensor is shown in Figure 8e. As shown in the figure, the COP flow sensor with the sensing element formed at the center of the tube was successfully fabricated.

**Figure 9:**
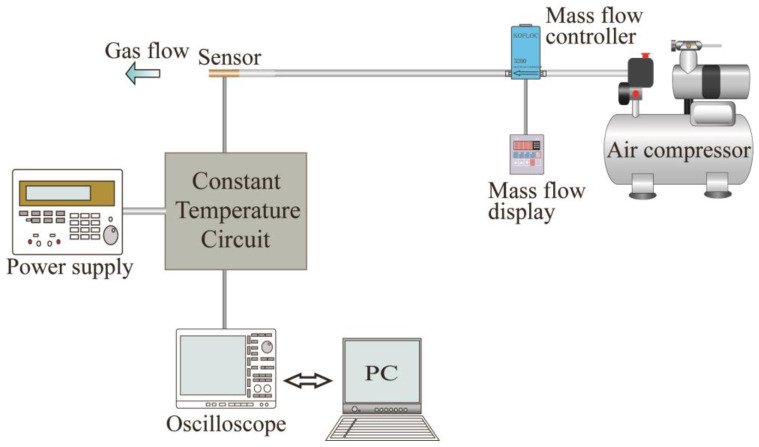
Experimental setup for characterization of the fabricated sensor.

**Figure 10:**
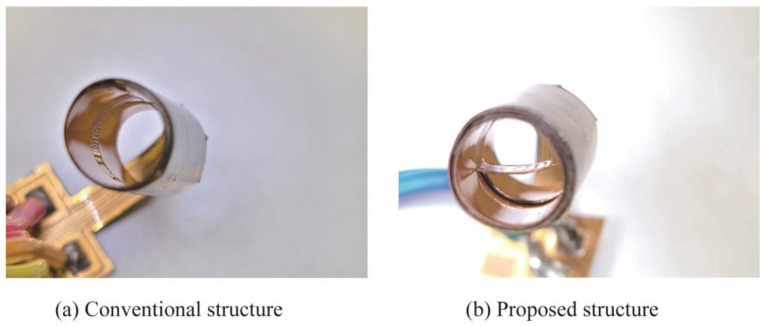
Photograph of a sensor with a conventional structure with the sensing element on the tube wall and the proposed sensor with the sensing element installed at the center of the tube.

Finally, the performance of the fabricated sensor was evaluated. The sensor output as a response to the amount of airflow through the cylinder was evaluated using a flow measurement equipment as schematically shown in Figure 9. The airflow was supplied by an air compressor. The supplied airflow was controlled using a commercially available mass flow controller (MODEL 3200, KOFLOC Corp., Kyoto, Japan) and passed through to the sensor. The experiment was performed with two types of flow sensors: the proposed one and the conventional one with the flow sensor installed on the wall surface, shown in Figure 10. The output signals from the sensors were processed by the same circuit and observed through it using an oscilloscope.

**Figure 11:**
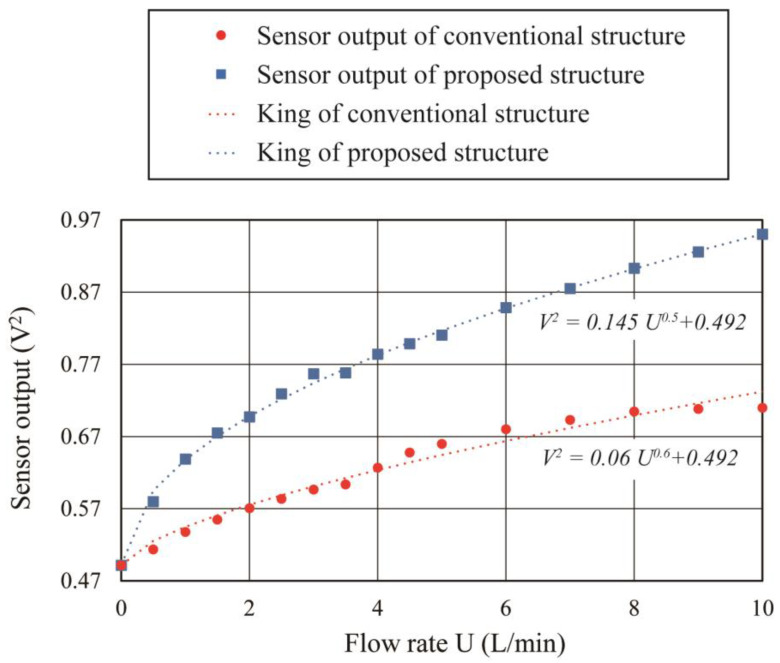
Flow characteristics of the conventional structure (red) and the proposed sensor (blue).

Figure 11 shows the measured sensor output in response to the supplied flow rate. The vertical axis in Figure 11 is the square of the sensor output which corresponds to the amount of thermal energy transferred during the sensing process. The sensor output of both types of sensors increased with the flow rate. The change in sensor output could be expressed by King’s equation, which shows the relationship of the amount of the heat transfer to the applied flow rate according to the hot wire anemometer principle [35]. The change in sensor output at 10 L/min airflow supply corresponds to 0.22 V^2^ for the conventional sensor and 0.46 V^2^ for the proposed structure, which shows almost twice the increase in sensitivity. In the future, we plan to confirm the flow direction detection function, and develop a sensor that also integrates a driving circuit on the COP substrate.

## 5. Conclusions

In this study, a tube-integrated MEMS flow sensor with the sensing element and electrical wiring on the same substrate was proposed and demonstrated. The sensor substrate itself was rolled to form a tube package. Furthermore, the sensing element was successfully placed at the center of the tube during the rolling process, where the flow velocity is maximum. For the proposed tube-integrated MEMS flow sensor, a COP substrate with a thin Cu layer on a polyimide film was used. The flow-sensing element was formed on a photosensitive polyimide layer patterned on the COP substrate. Then, the thin Cu layer was selectively etched through the holes in the photosensitive polyimide layer to form a cavity for thermal insulation and electrical wiring for the flow sensor. The structures fabricated on the COP substrate were incised and rolled to form a tube structure for the flow sensor. The sensing element was placed at the center of the tube during the rolling process, which is the place where the flow velocity is maximum. Finally, the flow characteristics of the fabricated sensor were evaluated, and the sensor sensitivity was approximately doubled in comparison to the conventional structure in which the sensing element is located on the tube wall.

## Figures and Tables

**Table 1:** Fluid simulation analysis conditions when the sensor is placed on the inner wall of in the center of the tube.

	Sensor installation location	Tube wall	Tube center
	Inlet flow velocity (m/s)	1.0	
	Outlet pressure	static	
Mesh	Number of meshes	150,000	150,000
Model geometry	Flow channel length (mm)	11.0	11.0
	Inner diameter (mm)	5.0	5.0
	Beam width (mm)	-	2.0
	Beam thickness (mm)	-	0.088

## Data Availability

Not applicable.
